# The CpG island methylator phenotype increases the risk of high-grade squamous intraepithelial lesions and cervical cancer

**DOI:** 10.1186/s13148-021-01224-0

**Published:** 2022-01-06

**Authors:** Jaqueline Loaeza-Loaeza, Berenice Illades-Aguiar, Oscar del Moral-Hernández, Yaneth Castro-Coronel, Marco A. Leyva-Vázquez, Roberto Dircio-Maldonado, Julio Ortiz-Ortiz, Daniel Hernández-Sotelo

**Affiliations:** 1 Laboratory of Cancer Epigenetics, School of Chemical and Biological Sciences, Autonomous University of Guerrero, Av. Lázaro Cárdenas S/N Col. Haciendita, 39070 Chilpancingo, Guerrero Mexico; 2 Laboratory of Molecular Biomedicine, School of Chemical and Biological Sciences, Autonomous University of Guerrero, Av. Lázaro Cárdenas S/N Col. Haciendita, 39070 Chilpancingo, Guerrero Mexico; 3 Laboratory of Cancer Virology, School of Chemical and Biological Sciences, Autonomous University of Guerrero, Av. Lázaro Cárdenas S/N Col. Haciendita, 39070 Chilpancingo, Guerrero Mexico; 4 Laboratory of Cytopathology and Histochemistry, School of Chemical and Biological Sciences, Autonomous University of Guerrero, Av. Lázaro Cárdenas S/N Col. Haciendita, 39070 Chilpancingo, Guerrero Mexico

**Keywords:** CpG, Methylation, Squamous intraepithelial lesion, Cervical cancer

## Abstract

**Background:**

High-risk human papillomavirus (HR-HPV) infection is the main cause of cervical cancer, but additional alterations are necessary for its development. Abnormal DNA methylation has an important role in the origin and dissemination of cervical cancer and other human tumors. In this work, we analyzed the methylation of eight genes (AJAP1, CDH1, CDH13, MAGI2, MGMT, MYOD1, RASSF1A and SOX17) that participate in several biological processes for the maintenance of cell normality. We analyzed DNA methylation by methylation-specific PCR (MSP) and HPV infection using the INNO‑LiPA genotyping kit in 59 samples diagnostic of normal cervical tissue (non-SIL), 107 low-grade squamous intraepithelial lesions (LSILs), 29 high-grade squamous intraepithelial lesions (HSILs) and 51 cervical cancers (CCs).

**Results:**

We found that all samples of LSIL, HSIL, and CC were HPV-positive, and the genotypes with higher frequencies were 16, 18, 51 and 56. In general, the genes analyzed displayed a significant tendency toward an increase in methylation levels according to increasing cervical lesion severity, except for the CDH13 gene. High CpG island methylator phenotype (CIMP) was associated with a 50.6-fold (95% CI 4.72–2267.3)-increased risk of HSIL and a 122-fold risk of CC (95% CI 10.04–5349.7).

**Conclusions:**

We found that CIMP high was significantly associated with HSIL and CC risk. These results could indicate that CIMP together with HR-HPV infection and other factors participates in the development of HSIL and CC.

**Supplementary Information:**

The online version contains supplementary material available at 10.1186/s13148-021-01224-0.

## Background

Cervical cancer remains a health problem in developing countries. Worldwide, there are approximately 529,800 new cases and 275,100 deaths every year [1]. The main cause of cervical cancer is persistent infection with HR-HPV [2]. This cancer evolves from the well-known precursor lesions LSIL and HSIL. Since not all cervical lesions with HR-HPV infections progress to cervical cancer, it is clear that additional events are necessary for progression [3, 4]. Epigenetic mechanisms are modifications that could have an important contribution to the progression of CC, particularly DNA methylation [5, 6].

DNA methylation frequently occurs at the cytosine-5-carbon adjacent to guanine (CpG), and CpG dinucleotides are abundant in regions denominated CpG islands [7]. DNA methylation plays important roles in genomic imprinting, embryonic development, chromatin structure and diseases such as cancer because it modulates the transcription of genes [8]. A fraction of the CpG islands is in gene promoters. In cancer, including cervical cancer, the hypermethylation of promoters of tumor suppressor and DNA repair genes is common [9–11]. Abnormal methylation participates in the initiation, transformation, and dissemination of cancer [8, 12]. Several studies have analyzed the abnormal methylation of genes that participate in cellular proliferation, apoptosis, differentiation, cell cycle, and cellular transformation during cervical carcinogenesis [13–17]. From these reports, it has been proposed that measurement of methylation could serve as a biomarker for early detection, diagnosis, and prognosis in cancer.

In this work, we analyzed the methylation of eight genes (AJAP1, CDH1, CDH13, MAGI2, MGMT, MYOD1, RASSF1A and SOX17) that participate in many biological processes. These genes were selected because they are involved in essential functions in the maintenance of cell normality and have CpG islands. Methylation of CpG islands in promoters decreases gene expression, and there are reports of hypermethylation in multiple types of cancer. The methylation of these eight genes has been evaluated in cervical cancer but has been evaluated individually; though their methylation has not been related to HPV, only a small number of samples have been analyzed, rather than systematically in normal cervical tissue (non-SIL), LSIL, HSIL, and CC. Therefore, in this work, we evaluated the methylation of eight genes and HPV genotypes in 58 non-SIL, 107 LSIL, 29 HSIL and 51 CC samples.

## Results

A total of 245 samples were included in this study: 58 non-SIL, 107 LSIL, 29 HSIL and 51 CC samples. Sociodemographic and sexual characteristics associated with the development of precancerous lesions and cervical cancer are shown in Table 1. The mean ages were 37.59 ± 10.97 years for non-SIL, 35.78 ± 11.94 years for LSIL, 39.37 ± 13.29 years for HSIL and 57.12 ± 13.18 for CC. The smoking status, alcohol consumption, parity, sexual age of onset and education years were statistically significant between non-SIL, LSIL, HSIL and CC.

**Table 1:** Sociodemographic and sexual conduct characteristics associated with precancerous lesions and cervical cancer

	Non-SIL		LSIL		HSIL		CC		*P*
	n = 58	%	n = 107	%	n = 29	%	n = 51	%	
Age (years)^a^	37.59 ± 10.97		35.78 ± 11.94		39.37 ± 13.29		57.12 ± 13.18		0.001^b^
Range	19–68		19–74		20–63		31–84		
Smoking status									
No	52	89.66	89	83.18	24	82.76	35	68.63	0.042^c^
Yes	6	10.34	15	14.02	3	10.34	10	19.61	
Unknown	0	0.0	3	2.80	2	6.90	6	11.76	
Alcohol consumption									
No	31	53.45	54	50.47	20	68.97	36	70.59	< 0.001^c^
Yes	27	46.55	50	46.73	7	24.14	9	17.65	
Unknown	0	0.0	3	2.80	2	6.90	6	11.76	
Parity									
None	33	55.93	62	57.94	6	20.69	2	3.92	< 0.001^c^
1–2	18	30.51	26	24.30	4	13.79	5	9.80	
3–5	6	10.17	16	14.95	7	24.14	17	33.33	
≥ 6	1	1.69	0	0.0	8	27.59	25	49.02	
Unknown	1	1.69	3	2.80	4	13.79	2	3.92	
Sexual age of onset									
< 16	3	5.08	12	11.21	5	17.24	16	31.37	< 0.001^c^
16–20	39	66.10	53	49.53	19	65.52	24	47.06	
> 20	16	27.12	40	37.38	3	10.34	8	15.69	
Unknown	1	1.69	2	1.87	2	6.90	3	5.88	
Number of life time sexual partners									
1–2	48	81.36	75	70.09	22	75.86	35	68.63	0.056^c^
≥ 3	10	16.95	28	26.17	4	13.79	9	17.65	
Unknown	1	1.69	4	3.74	3	10.34	7	13.73	
Education (years)									
0	0	0.0	3	2.80	7	24.14	19	37.25	< 0.001^c^
6	2	3.39	7	6.54	7	24.14	22	43.14	
9	4	6.78	8	7.48	3	10.34	0	0.0	
12	5	8.47	15	14.02	1	3.45	5	9.80	
≥ 13	48	81.36	70	65.42	9	31.03	1	1.96	
Unknown	0	0.0	4	3.74	2	6.90	4	7.84	

CC cervical cancer, HSIL high-grade squamous intraepithelial lesion, LSIL low-grade squamous intraepithelial lesion, Non-SIL negative for squamous intraepithelial lesion

^a^Expressed as mean ± standard deviation

^b^Kruskal-Wallis

^c^chi-squared

High-risk HPVs are the cause of cervical cancer. We determined the prevalence of single, multiple, and mixed HPV infections in the samples included in this study (Table 2). The prevalence rates of HPV infection were 49.15% in non-SIL and 100% in LSIL, HSIL, and CC. Single infection with HR-HPV was detected in 23.73% of non-SILs, 41.12% of LSILs, 34.48% of HSILs and 60.78% of CCs, while multiple infections with HR-HPV were detected in 3.39% of non-SILs, 19.63% of LSILs, 24.14% of HSILs and 21.57% of CCs. Mixed HPV infections were more frequent, with HR and low-risk (LR) infections in 10.17% of non-SILs, HR and probably high-risk (PHR) infections in 21.5% of LSILs, HR and PHR infections in 13.79% of HSILs and HR and LR infections in 9.8% of CCs. Additionally, in Additional file 1: Table S1, we show the prevalence of all HPV genotypes found in this study. HPV16, 18, 51 and 56 were the genotypes more frequently detected.

**Table 2:** Prevalence of single, multiple and mix HPV infection in Non-SIL, LSIL, HSIL and, CC

	Non-SIL		LSIL		HSIL		CC	
	n = 59	%	n = 107	%	n = 29	%	n = 51	%
HPV negative	30	50.85	0	0.0	0	0.0	0	0.0
HPV positive	29	49.15	107	100	29	100	51	100
*Single infection*								
HR	14	23.73	44	41.12	10	34.48	31	60.78
PHR	3	5.08	3	2.80	1	3.45	0	0.0
LR	0	0.0	3	2.80	0	0.0	0	0.0
UR	0	0.0	2	1.87	0	0.0	0	0.0
Total	17	28.81	52	48.59	11	37.93	31	60.78
*Multiple HPV infection*								
HR	2	3.39	21	19.63	7	24.14	11	21.57
*Mix HPV infection*								
HR and PHR	2	3.39	23	21.50	4	13.79	1	1.96
HR and LR	6	10.17	5	4.67	3	10.34	5	9.80
HR and UR	1	1.69	0	0.0	0	0.0	0	0.0
PHR and LR	0	0.0	0	0.0	0	0.0	1	1.96
PHR and UR	0	0.0	0	0.0	1	3.45	1	1.96
HR, PHR and LR	1	1.69	6	5.61	2	6.90	0	0.0
HR, PHR and UR	0	0.0	0	0.0	0	0.0	1	1.96
HR, PHR, LR and UR	0	0.0	0	0.0	1	3.45	0	0.0
Total	10	16.94	34	31.78	11	37.93	9	17.64

CC cervical cancer, HSIL high-grade squamous intraepithelial lesion, LSIL low-grade squamous intraepithelial lesion, Non-SIL negative for squamous intraepithelial lesion, HR high risk, PHR probably high risk, LR low risk, UR indeterminate risk

**Fig. 1:**
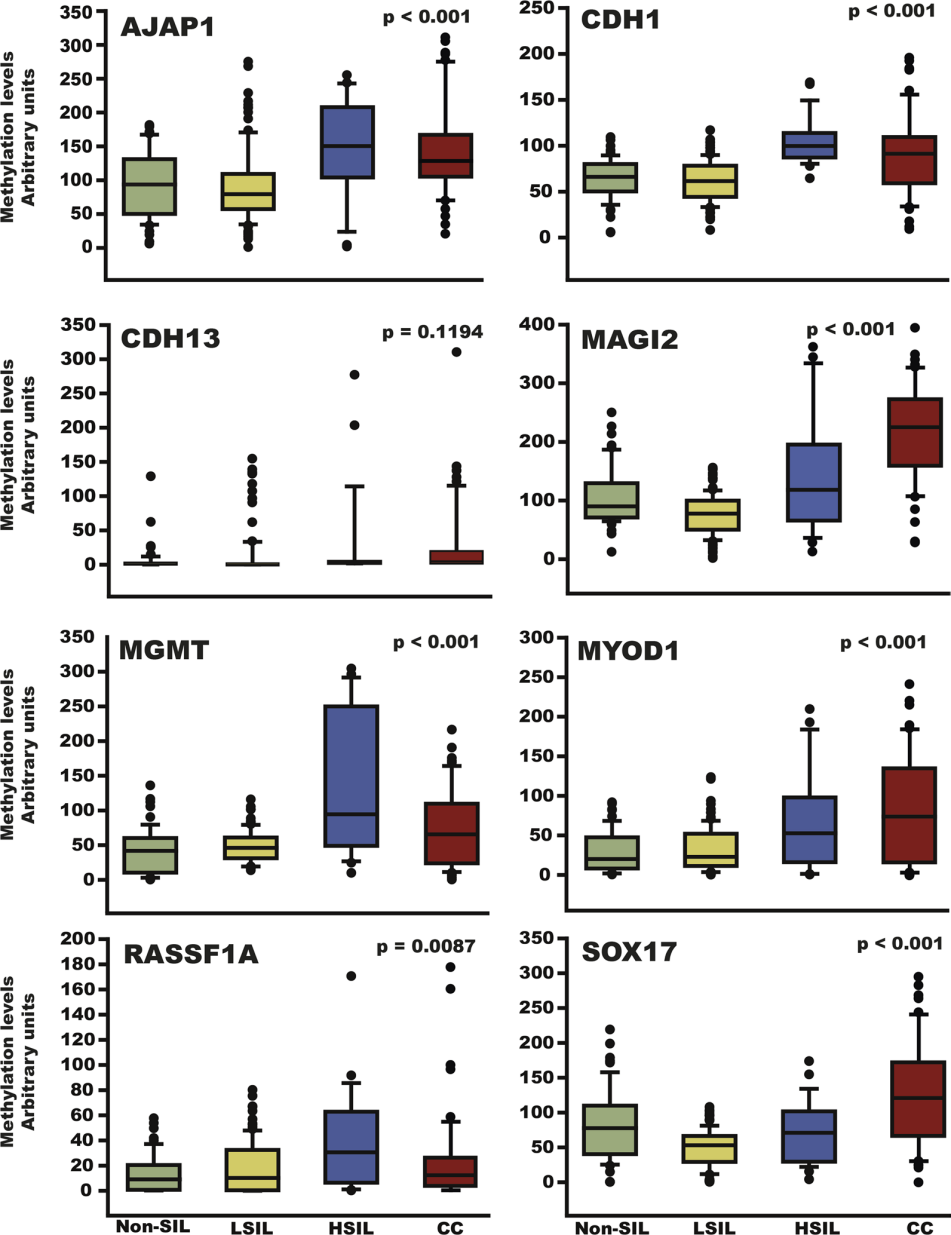
Analysis of the methylation levels of the *AJAP1, CDH1, CDH13, MAGI2, MGMT, MYOD1, RASSF1A* and *SOX17* genes in cervical tissue. Methylation was analyzed in 58 samples negative for squamous intraepithelial lesions (non-SILs), 107 low-grade squamous intraepithelial lesions (LSILs), 29 high-grade squamous intraepithelial lesions (HSILs) and 51 cervical cancers (CCs). The p values were calculated using the Kruskal–Wallis test

**Fig. 2:**
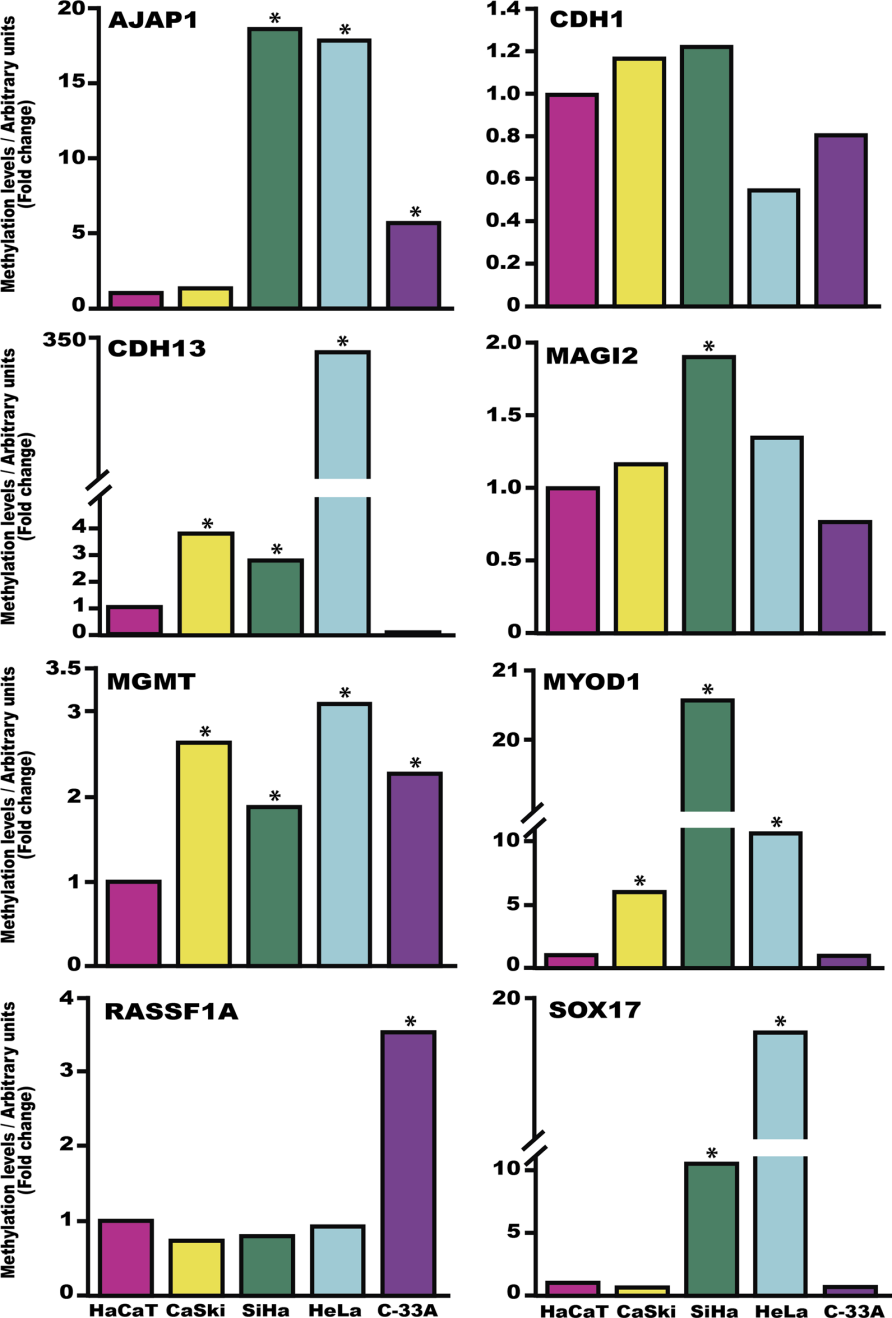
Analysis of the methylation levels of the *AJAP1, CDH1, CDH13, MAGI2, MGMT, MYOD1, RASSF1A* and *SOX17* genes in cervical cancer cell lines. The data are presented as fold changes in the cancer cell line relative to the HaCaT cell line. *p < 0.05

At the present time, there is evidence that DNA methylation is involved in the genesis and progression of cervical cancer. In this work, we analyzed the DNA methylation status of eight genes (AJAP1, CDH1, CDH13, MAGI2, MGMT, MYOD1, RASSF1A and SOX17) in 245 cervical tissue samples (Fig. 1). In general, the genes displayed a significant tendency toward an increase in methylation levels according to increasing cervical lesion severity, except for the CDH13 gene. Additionally, we analyzed the DNA methylation level and expression of these genes in cervical cancer samples (TCGA data; Additional file 3: Fig. S2). In this data set, we found methylation and lower expression in AJAP1, SOX17, CDH1, and RASSF1A genes. Furthermore, we analyzed the DNA methylation level and mRNA expression levels of these genes in four cervical cancer cell lines as a reference to the HaCaT cell line (Fig. 2 and Additional file 4: Fig. S3). AJAP1 methylation was significantly more prevalent in the SiHa, HeLa and C-33A cell lines. MYOD1 and CDH13 methylation was significantly more prevalent in CaSki, SiHa and HeLa cell lines. Only MGMT methylation was significantly more prevalent in the four cell lines, while CDH1 methylation was not significantly prevalent in any of the cell lines. SOX17 methylation was significantly more prevalent in the SiHa and HeLa cell lines, and RASSF1A and MAGI2 methylations were only observed in C-33A and SiHa cells, respectively (Fig. 2). In general, mRNA expression levels are lower in cervical cancer cells lines than in HaCaT cells, except for SOX17 and MYOD1 in SiHa cells, MGMT in C-33A cells and AJAP1, SOX17 and MYOD1 in HeLa cells (Additional file 4: Fig. S3).

Using densitometric analysis, we semiquantified the methylation levels of all the samples by MSP. The methylation level of each gene was categorized into three groups, unmethylated, methylated and hypermethylated, and an arbitrary value was assigned to each category: 0, 1 and 2, respectively. Additional file 5: Fig. S4 and Additional file 6: Fig. S5 show the methylation levels of eight genes in non-SIL, LSIL, HSIL, CC samples and cell lines.

**Fig. 3:**
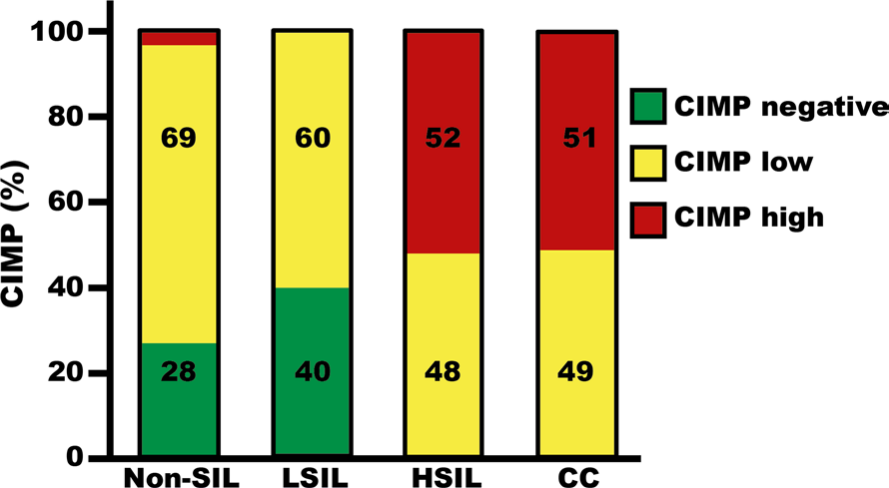
CpG island methylation phenotype (CIMP) frequencies in non-SIL, LSIL, HSIL and CC

Previously, the presence of the CIMP has been reported in several types of human cancer. This phenotype is characterized by the existence of commonly methylated genes that can influence different aspects of cancer biology. For CIMP analysis, the arbitrary values of the three categories were used (unmethylated 0, methylated 1 and hypermethylated 2). Thus, the sum of arbitrary values of the eight genes by the patient was used to define CIMP categories: CIMP negative (0 to 1), CIMP low (2 to 5) and CIMP high (> 6). The CIMP analysis of non-SIL, LSIL, HSIL, and CC samples is shown in Fig. 3. CIMP high was found mainly in CC and HSIL, and CIMP negative was found in non-SIL and LSIL.

The CIMP status represents the sum of abnormal methylation of the eight genes analyzed by sample. Therefore, we evaluated the CIMP status and the risk of LSIL, HSIL, and CC development (Table 3). CIMP high was associated with a 50.6-fold (95% CI 4.72–2267.3)-increased risk of HSIL and a 122-fold risk of CC (95% CI 10.04–5349.7). Given that the CIMP statuses between non-SIL and LSIL or HSIL and CC are very similar (Fig. 3), we analyzed the risk of this mode. CIMP high was associated with a 158.6-fold (95% CI 28.9–1471.41) increased risk of HSIL + CC. Additionally, Additional file 1: Tables S2 to S9 show the methylation status of each gene and the risk of LSIL, HSIL, and CC development.

**Table 3:** CIMP status and risk of cervical lesion

	CIMP		OR	CI	*P*
	Negative *n*	High *n*			
Non-SIL	16	2	1		
LSIL	43	1	0.19	0.003–3.92	0.141
HSIL	1	15	**120**	7.95–5342.9	0.0000
CC	1	25	**200**	13.75–8683.9	0.0000
Non-SIL	16	2	1		
HSIL and CC	2	40	**160**	16.64–1960.8	0.0000
Non-SIL and LSIL	59	3	1		
HSIL and CC	2	40	**393.3**	52.9–4040.78	0.0000

	Negative + low *n*	High *n*			
Non-SIL	57	2	1		
LSIL	107	1	0.27	0.004–5.26	0.251
HSIL	14	15	**30.5**	5.77–290-09	0.0000
CC	26	25	**27.4**	5.91–248.19	0.0000

Significant values for associations are indicated in bold

CIMP, CpG island methylation phenotype; OR, odd ratio; CI, confidence interval; CC, cervical cancer; HSIL, high-grade squamous intraepithelial lesion; LSIL, low-grade squamous intraepithelial lesion; Non-SIL, negative for squamous intraepithelial lesion

## Discussion

It is known that persistent infection with HR-HPV is the cause of cervical cancer [2]. However, additional alterations are necessary for the development of cervical lesions and cervical cancer [4]. Abnormal DNA methylation has an important role from genesis to the dissemination of cervical cancer and other human tumors [12, 18]. In this study, we evaluated the DNA methylation of eight genes, identified and genotyped DNA-HPV, and analyzed the CIMP status and the risk of LSIL, HSIL, and CC development.

CIMP-positive tumors are a class of cancers that have concurrent hypermethylation of multiple genes, and such genes are involved in cellular transformation, proliferation, migration, and invasion, which are key functions in the genesis of human cancer [19, 20]. In our work, CIMP high represents a subclass of patients with methylation or hypermethylation in most of the genes analyzed, and it is likely that such genes are decreased or not expressed. In fact, several studies have reported DNA methylation and decreases or a lack of expression of these genes in several types of human cancer [6, 13, 21]. Furthermore, in general, the mRNA levels of these genes were lower in cervical cancer than in normal cervical tissue (TCGA data; Additional file 7: Fig. S6). We found that CIMP high was significantly associated with an increased risk of HSIL (OR = 50.6) and CC (OR = 122). In agreement with our results, two studies have reported that CIMP high is significantly associated with HSIL and CC development [12, 21]. Biologically, this result can be explained by the individual contribution of each abnormally methylated gene in the molecular alterations that cause HSIL and CC development.

The analyzed genes in this work (SOX17, MYOD1, MAGI2, CDH1, AJAP1, MGMT, CDH13, and RASSF1A) participate in essential biological processes to maintain cell normality, and there is sufficient evidence for their protective role against the development of cancer. For example, SOX17 is a transcription factor involved in cellular reprogramming and has an antagonist role in WNT signaling [22]. There is evidence of its silencing by methylation in several types of human cancer [23–25]. MYOD1 is a master regulator of differentiation in muscle cells [26]. Abnormal promoter methylation is frequent in cervical cancer and other human cancers [27–29]. MAGI2 belongs to the membrane-associated guanylate kinase superfamily and is an important part of tight junction proteins in epithelial cells [30]. MAGI2, beta-catenin and other proteins participate in migration and proliferation in some cell types [21]. Abnormal expression and hypermethylation in this gene are common events in different types of cancer [31, 32]. In our work, the hypermethylation of the SOX17, MYOD1 and MAGI2 genes was significantly associated with CC development. The CDH1 gene encodes E-cadherin, which is a transmembrane glycoprotein involved in cell–cell adhesion and is key for controlling cell maturation and movement [33]. CDH1 is considered a tumor suppressor gene and is frequently silenced by the methylation of its promoter in cervical cancer and other human tumors [13, 33, 34]. AJAP1 is a transmembrane protein that is an important part of cell–cell and cell-extracellular matrix interactions [35]. This gene has been considered a tumor suppressor and is epigenetically silenced by methylation in cervical cancer, glioma, esophageal squamous cell carcinoma, lung cancer and endometrial carcinoma [21, 35–38]. We found that the hypermethylation of CDH1 and AJAP1 was significantly associated with HSIL and CC development. MGMT encodes a DNA repair protein that is removed by direct repair of oxygen 6-methylguanine lesions [39]. Lack of expression and promoter hypermethylation are frequent in human cancers, including cervical cancer [40, 41]. We found that the hypermethylation of MGMT was significantly associated with HSIL development. RASSF1A is a tumor suppressor gene that encodes scaffold proteins [42]. Additionally, hypermethylation and lack of expression of this gene are frequent in cancer [43, 44]. In our work, the methylation level of RASSF1A increased according to cervical lesion severity.

CDH13 is an atypical cadherin since it lacks the transmembrane and cytoplasmic domains to mediate its functions through its signaling properties [45]. This gene is considered a tumor suppressor, and its inactivation by hypermethylation is common in colorectal cancer and human lung adenocarcinoma cell lines [46–48]. Although CDH13 has a clear function in cancer, we did not find that its methylation was associated with HSIL and CC development. In agreement with our results, there is work that shows low-frequency or absence of methylation in this gene [13, 21, 49]. There is no evidence of CDH13 methylation in cervical cancer. We believe that this is because DNMT3B and 3A (de novo methylases) does not show an affinity for the promoter of this gene and that the decrease in its expression is explained by other components of epigenetic regulation [8, 10, 20].

Four of the genes (SOX17, MAGI2, AJAP1 and CDH1) that we analyzed and that were significantly associated with HSIL, and CC development play essential roles in the WNT/β-catenin signaling pathway [21, 50]. This signaling pathway is crucial in embryogenesis and cell differentiation, and abnormalities in this pathway are common in human cancers, including cervical cancer [51–53].

We do not show concrete evidence that this panel of genes is involved in the initiation, development and progression of HSIL or CC. We only show epidemiological evidence of the methylation role and the risk of development HSIL and cervical cancer. Another limitation of our study is that unfortunately, we did not analyze the expression of the 8 genes in the included samples. Instead, we analyzed the expression of these genes in cervical cancer cell lines and databases. Although there is concordance between the expression and methylation data of the cell lines and the analyzed samples, we must take these similarities with caution. The cell lines are homogeneous and do not reflect the complexity of cervical tumor tissue.

## Conclusions

We found that CIMP high was significantly associated with HSIL and CC development. This result could indicate that CIMP in precancerous lesions and cervical cancer participates in its genesis together with HR-HPV infection. Additionally, the SOX17, MAGI2, AJAP1, MYOD1, MGMT, and CDH1 genes can be used as biomarkers in HSIL and CC development.

## Methods

### DNA samples

This study was conducted with 245 DNA samples from the Sample Bank of the Laboratory of Biomedicina Molecular, Universidad Autónoma de Guerrero, México. The population consisted of 58 samples diagnostic of non-SIL, 107 LSIL, 29 HSIL and 51 CC. The diagnosis of non-SIL and LSIL was performed by cytomorphological examination through the Papanicolaou test and diagnosis of HSIL and CC by histological diagnosis, according to the International Federation of Gynecology and Obstetrics. The Bioethics and Research Committee of the Institute approved the study (UAGro-IECan 04/18/2016), according to the ethical guidelines of the 2008 Helsinki Declaration.

### Cell culture

Human cervical carcinoma (C-33A, SiHa, CaSki and HeLa) and human skin keratinocyte (HaCaT) cell lines were obtained from the American Type Culture Collection (ATCC, USA). Cells were grown at 37 °C in a 5% CO_2_ atmosphere with DMEM and F-12 1:1 (Sigma–Aldrich, St. Louis, MO) supplemented with 10% fetal bovine serum, 100 U/ml penicillin and 100 µg/ml streptomycin.

### HPV detection and genotyping

Genomic DNA of clinical samples was extracted from cervical cells by the phenol chloroform method. Genomic DNA of cell lines was extracted using the Wizard® Genomic DNA Purification Kit (Promega; Madison, WI, USA) according to the manufacturer´s instructions. HPV detection and genotyping were performed with an INNO‑LiPA genotyping kit (Fujirebio Europe, Gent, Belgium) without amendment and according to the manufacturer's protocol.

### Methylation-specific PCR

Methylation analysis of the AJAP1, CDH1, CDH13, MAGI2, MGMT, MYOD1, RASSF1A and SOX17 genes was performed by MSP and densitometric analysis was performed with ImageJ software (NIH; Bethesda, Maryland, USA). Primer sequences are shown in Table 4. Briefly, 2 μg of genomic DNA was modified using the EZ DNA Methylation-Gold™ Kit (Zymo Research; Irvine, CA, USA). PCR was performed using Amplitaq Gold Master Mix (Applied Biosystems; Foster City, CA, USA) according to the manufacturer’s protocol. Amplification conditions were as follows: denaturation, 95 °C for 10 min; 30 to 35 cycles of amplification: 30 s at 95 °C, 30 s at 60 °C and 30 s at 72 °C; and a final extension of 72 °C for 10 min. DNA from human leukocytes methylated in vitro with M.SssI (New England Biolabs, Inc., Ipswich, USA) was used as a positive control, and reactions without DNA were used as a negative control. Additional controls were included, selection was made from information collected from cell lines of different tissues and pathological states in the genome browser. RRBS and Methyl 450 K Array methylation dates were charge for the CpG island of GAPDH (unmethylated) and OXT (Methylated) genes. Furthermore, OXT has been used as a control methylated DNA by MeDIP assay [54] (Additional file 2: Fig. S1).

**Table 4:** Primer sequences

Gene	5′ to 3′	Tm °C	
AJAP1	M	F: TTTGGTAGAGTTTTTCGATTCGGTAGC R: ACCGAAACTCCGCGCCGATAA	60
	U	F: TTTGGTAGAGTTTTTTGATTTGGTAGT R: CCAAAACTCCACACCAATAA	55
CDH1	M	F: TTAGGTTAGAGGGTTATCGCGT R: TAACTAAAATTCACCTACCGAC	57
	U	F: TAATTTTAGGTTAGAGGGTTATTGT R: CACAACCAATCAACAACACA	55
CDH13	M	F: TCGCGGGGTTCGTTTTTCGC R: GACGTTTTCATTCATACACGCG	57
	U	F: TTGTGGGGTTTGTTTTTTGT R: ACATTTTCATTCATACACACA	53
MAGI2	M	F: CGTAGAGTTCGAGATGTGGTATTAGGC R: AAACTCCTATACGAAAAAAACGCGCTA	60
	U	F: TGTAGAGTTTGAGATGTGGTATTAGGT R: AACTCCTATACAAAAAAAACACACTA	55
MGMT	M	F: TTTCGACGTTCGTAGGTTTTCGC R: GCACTCTTCCGAAAACGAAACG	59
	U	F: TTTGTGTTTTGATGTTTGTAGGTTTTTGT R: AACTCCACACTCTTCCAAAAACAAAACA	59
MYOD1	M	F: GACGGTTTTCGACGGTTT R: GCCCGAAACCGAATACAC	56
	U	F: ATTTGATGGTTTTTGATGGTTT R: CACACACATACTCATCCTCACA	57
RASSF1A	M	F: GTGTTAACGCGTTGCGTATC R: AACCCCGCGAACTAAAAACGA	65
	U	F: TTTGGTTGGAGTGTGTTAATGTG R: CAAACCCCACAAACTAAAAACAA	60
SOX17	M	F: GGAGATTCGCGTAGT TTTCG R: AACCCGACCATCACCGCG	60
	U	F: GGAGATTTGTGTAGT TTTTG R: ACCCAACCATCACCACA	55
GAPDH	M	F: GAGAAAGTAGGGTTCGGTTATTAGC R: AAAAACGAAACGAAAAACTACGA	55
OXT	M	F: ATAAAAAGGTTAGGTCGGAGAGATC R: AAATATAACAAACGAAAATCAACGC	52

M, methylated; U, unmethylated; F, forward; R, reverse

### Semiquantification of methylation levels

We performed semiquantification of methylation levels by densitometry analysis with ImageJ. The quantitative values of each sample and of each gene were obtained, and the minimum and maximum values of each sample group (non-SIL, LSIL, HSIL and CC) were calculated tertiles. With these data, we established three categories: unmethylated (first tertile), methylated (second tertile) and hypermethylated (third tertile). In addition, for CpG island methylation phenotype (CIMP) analysis, we assigned an arbitrary value to each category: 0 for unmethylated, 1 for methylated and 2 for hypermethylated. Thus, with the sum of arbitrary values of the eight genes by each patient, CIMP categories were established: CIMP negative (0 to 1), CIMP low (2 to 5) and CIMP high (> 6).

### Statistical analysis

All results are expressed as the mean ± standard deviation. The chi-squared and Kruskal–Wallis tests were used to determine the differences between non-SIL, LSIL, HSIL and CC. The associations between CIMP status and the risk of developing CC, HSIL, and LSIL were estimated by odds ratios (ORs) using the STATA 10.0 software package (StataCorp, College Station, TX, USA). Ninety-five percent confidence intervals (95% CIs) and *p* values are reported for the OR.

## Supplementary Information

**Additional file 1**. **Table S1**. Prevalence of genotypes HPV in single, multiple and mix HPV infection in normal cervix, precancerous lesions and cervical cancer; **Table S2**. Methylation status of CDH1 and risk of cervical lesion; **Table S3**. Methylation status of AJAP1 and risk of cervical lesion; **Table S4**. Methylation status of MAGI2 and risk of cervical lesion; **Table S5**. Methylation status of MYOD1 and risk of cervical lesion; **Table S6**. Methylation status of SOX17 and risk of cervical lesion; **Table S7**. Methylation status of MGMT and risk of cervical lesion; **Table S8**. Methylation status of RASSF1A and risk of cervical lesion; **Table S9**. Methylation status of CDH13 and risk of cervical lesion.

**Additional file 2: Fig. S1**. Analysis of the methylation levels of control genes in cervical tissue. Methylation was analyzed in 5 samples negative for squamous intraepithelial lesions (non-SILs), 5 low-grade squamous intraepithelial lesions (LSILs), 5 high-grade squamous intraepithelial lesions (HSILs) and 5 cervical cancers (CCs). a) MSP products in agarose gels for methylated *GAPDH* (168 bp) and methylated *OXT *(131 bp). b) Densitometry analysis from *GAPDH* and *OXT* methylation. The data are presented as mean ± standard deviation at 5 samples in each group.

**Additional file 3: Fig. S2**. Methylation and expression heatmaps of *AJAP1, SOX17, CDH13, MAGI2, MGMT, CDH1, RASSF1*, and *MYOD1* genes in public database (Cervical Squamous Cell Carcinoma and Endocervical Adenocarcinoma, TCGA, Firehose Legacy). a) Methylation data of 309 patients. b) Expression level of analyzed genes. The yellow box shows a representative patient’s group for *AJAP1* gene with high methylation levels and low expression levels.

**Additional file 4: Fig. S3**. Analysis of the expression levels of the *AJAP1, CDH13, MAGI2, SOX17, MGMT*, and *MYOD1* genes in cervical cancer cell lines. The dates are presented as the fold change in cancer cell line relative to HaCaT cell line. *p<0.05

**Additional file 5: Fig. S4**. Analysis of the methylation levels of the *AJAP1, CDH1, CDH13, MAGI2, MGMT, MYOD1, RASSF1A* and *SOX17* genes in each of the non-SIL and LSIL samples. The sum of arbitrary values of methylation and CIMP status is shown.

**Additional file 6: Fig. S5**. Analysis of the methylation levels of the *AJAP1, CDH1, CDH13, MAGI2, MGMT, MYOD1, RASSF1A* and *SOX17* genes in each of the HSIL and CC samples. The sum of arbitrary values of methylation and CIMP status is shown.

**Additional file 7: Fig. S6**. Expression of analyzed genes in TCGA dataset. The mRNA levels of analyzed genes were investigated in normal and cancer samples. Cancer included cervical squamous cell carcinoma and endocervical adenocarcinoma tumor samples. *p<0.05

## Data Availability

Not applicable.
