# Supplementary material for: The CpG island methylator phenotype increases the risk of high-grade squamous intraepithelial lesions and cervical cancer
Source: Clin Epigenetics. 2022 Jan 6;14:4. doi: 10.1186/s13148-021-01224-0 (PMC8740093; doi:10.1186/s13148-021-01224-0)
Supplement: Supplementary file 1 — Additional file 1. Table S1. Prevalence of genotypes HPV in single, multiple and mix HPV infection in normal cervix, precancerous lesions and cervical cancer; Table S2. Methylation status of CDH1 and risk of cervical lesion; Table S3. Methylation status of AJAP1 and risk of cervical lesion; Table S4. Methylation status of MAGI2 and risk of cervical lesion; Table S5. Methylation status of MYOD1 and risk of cervical lesion; Table S6. Methylation status of SOX17 and risk of cervical lesion; Table S7. Methylation status of MGMT and risk of cervical lesion; Table S8. Methylation status of RASSF1A and risk of cervical lesion; Table S9. Methylation status of CDH13 and risk of cervical lesion. [file 13148_2021_1224_MOESM1_ESM.docx]

| Table S1. Prevalence of genotypes HPV in single, multiple and mix HPV infection in normal cervix, precancerous lesions and cervical cancer | | | | | | | | |
| --- | --- | --- | --- | --- | --- | --- | --- | --- |
|  | Non-SIL | | LSIL | | HSIL | | CC | |
|  | n=59 | % | n=107 | % | n=29 | % | n=51 | % |
| HPV negative | 30 | 50.85 | 0 | 0.0 | 0 | 0.0 | 0 | 0.0 |
| Single HPV infection |  |  |  |  |  |  |  |  |
| 16 HR | 3 | 5.08 | 10 | 9.35 | 4 | 13.79 | 16 | 31.37 |
| 18 HR | 1 | 1.69 | 4 | 3.74 | 2 | 6.90 | 5 | 9.80 |
| 31 HR | 1 | 1.69 | 2 | 1.87 | 1 | 3.45 | 1 | 1.96 |
| 33 HR | 0 | 0.0 | 0 | 0.0 | 0 | 0.0 | 2 | 3.92 |
| 39 HR | 1 | 1.69 | 2 | 1.87 | 0 | 0.0 | 1 | 1.96 |
| 45 HR | 1 | 1.69 | 2 | 1.87 | 1 | 3.45 | 3 | 5.88 |
| 51 HR | 3 | 5.08 | 1 | 0.93 | 1 | 3.45 | 0 | 0.0 |
| 52 HR | 2 | 3.39 | 4 | 3.74 | 0 | 0.0 | 2 | 3.92 |
| 56 HR | 2 | 3.39 | 14 | 13.08 | 0 | 0.0 | 0 | 0.0 |
| 58 HR | 0 | 0.0 | 1 | 0.93 | 1 | 3.45 | 0 | 0.0 |
| 59 HR | 0 | 0.0 | 4 | 3.74 | 0 | 0.0 | 1 | 1.96 |
| 53 PHR | 3 | 5.08 | 0 | 0.0 | 0 | 0.0 | 0 | 0.0 |
| 66 PHR | 0 | 0.0 | 3 | 2.80 | 1 | 3.45 | 0 | 0.0 |
| 44 LR | 0 | 0.0 | 2 | 1.87 | 0 | 0.0 | 0 | 0.0 |
| Multiple HPV infection |  |  |  |  |  |  |  |  |
| 16,18 HR | 0 | 0.0 | 0 | 0.0 | 0 | 0.0 | 2 | 3.92 |
| 16,39 HR | 0 | 0.0 | 2 | 1.87 | 0 | 0.0 | 1 | 1.96 |
| 16,52 HR | 0 | 0.0 | 1 | 0.93 | 1 | 3.45 | 2 | 3.92 |
| 16,56 HR | 1 | 1.69 | 0 | 0.0 | 1 | 3.45 | 0 | 0.0 |
| 31,33 HR | 0 | 0.0 | 4 | 3.74 | 3 | 10.34 | 2 | 3.92 |
| 39,52 HR | 0 | 0.0 | 2 | 1.87 | 0 | 0.0 | 0 | 0.0 |
| 45,52 HR | 0 | 0.0 | 1 | 0.93 | 0 | 0.0 | 1 | 1.96 |
| 52,56 HR | 0 | 0.0 | 2 | 1.87 | 0 | 0.0 | 0 | 0.0 |
| Mix HPV infection |  |  |  |  |  |  |  |  |
| 16,53 HR,PHR | 0 | 0.0 | 3 | 2.80 | 0 | 0.0 | 0 | 0.0 |
| 45,66 HR,PHR | 0 | 0.0 | 1 | 0.93 | 1 | 3.45 | 0 | 0.0 |
| 52,53 HR,PHR | 1 | 1.69 | 1 | 0.93 | 0 | 0.0 | 0 | 0.0 |
| 56,53 HR,PHR | 0 | 0.0 | 1 | 0.93 | 1 | 3.45 | 0 | 0.0 |
| 51,52,66 HR,PHR | 0 | 0.0 | 2 | 1.87 | 0 | 0.0 | 0 | 0.0 |
| 16,6 HR,LR | 1 | 1.69 | 1 | 0.93 | 1 | 3.45 | 1 | 1.96 |
| 16,11 HR,LR | 0 | 0.0 | 1 | 0.93 | 0 | 0.0 | 2 | 3.92 |
| 16,68,6 HR,LR | 2 | 3.39 | 0 | 0.0 | 0 | 0.0 | 0 | 0.0 |
| CC cervical cancer, HSIL high-grade squamous intraepithelial lesion, LSIL low-grade squamous intraepithelial lesion, Non-SIL negative for squamous intraepithelial lesión, HR high risk, PHR probably high risk, LR low risk  Single HPV: infection with one genotype (are shown the genotypes more prevalent by risk group), Multiple HPV: infection with two genotypes of the same risk group (are shown the genotypes more prevalent by risk group), Mix HPV: infection with genotypes of diferent risk group (are shown the combinations more prevalent) | | | | | | | | |

| Table S2. Methylation status of CDH1 and risk of cervical lesion | | | | | |
| --- | --- | --- | --- | --- | --- |
|  | Unmethylated  n | Hypermethylated  n | OR | CI | *P* |
| Non-SIL | 33 | 1 | 1 |  |  |
| LSIL | 68 | 1 | 0.485 | 0.006-39.23 | 0.605 |
| HSIL | 1 | 4 | **132** | 4.66-6662.9 | 0.0000 |
| CC | 17 | 7 | **13.6** | 1.48-630.32 | 0.0043 |
|  |  |  |  |  |  |
| Non-SIL | 33 | 1 | 1 |  |  |
| HSIL and CC | 18 | 11 | **20.16** | 2.45-893.41 | 0.0004 |
|  |  |  |  |  |  |
| Non-SIL and LSIL | 101 | 2 | 1 |  |  |
| HSIL and CC | 18 | 11 | **36.86** | 5.79-296.96 | 0.0000 |
|  | Methylated +  Unmethylated  n | Hypermethylated  n |  |  |  |
| Non-SIL | 59 | 1 | 1 |  |  |
| CC | 44 | 7 | **9.38** | 1.12-429.96 | 0.0144 |
| OR odd ratio, CI confidence interval, CC cervical cancer, HSIL high-grade squamous intraepithelial lesion, LSIL low-grade squamous intraepithelial lesion, Non-SIL negative for squamous intraepithelial lesion; Significant values for associations are indicated in bold. | | | | | |

| Table S3. Methylation status of AJAP1 and risk of cervical lesion | | | | | |
| --- | --- | --- | --- | --- | --- |
|  | Unmethylated  n | Hypermethylated  n | OR | CI | *P* |
| Non-SIL | 36 | 1 | 1 |  |  |
| LSIL | 80 | 5 | 2.25 | 0.24-109.31 | 0.455 |
| HSIL | 7 | 7 | **36** | 3.40-1664.7 | 0.0000 |
| CC | 12 | 8 | **24** | 2.59-1093.2 | 0.0002 |
|  |  |  |  |  |  |
| Non-SIL | 36 | 1 | 1 |  |  |
| HSIL and CC | 19 | 15 | **28.42** | 3.66-1228.5 | 0.0000 |
|  |  |  |  |  |  |
| Non-SIL and LSIL | 116 | 5 | 1 |  |  |
| HSIL and CC | 19 | 15 | **18.31** | 5.38-70.07 | 0.0000 |
|  | Methylated +  Unmethylated  n | Hypermethylated  n |  |  |  |
| Non-SIL | 59 | 1 | 1 |  |  |
| CC | 43 | 8 | **10.97** | 1.36-495.03 | 0.007 |
| OR odd ratio, CI confidence interval, CC cervical cancer, HSIL high-grade squamous intraepithelial lesion, LSIL low-grade squamous intraepithelial lesion, Non-SIL negative for squamous intraepithelial lesion; Significant values for associations are indicated in bold. | | | | | |

| Table S4. Methylation status of MAGI2 and risk of cervical lesion | | | | | |
| --- | --- | --- | --- | --- | --- |
|  | Unmethylated  n | Hypermethylated  n | OR | CI | *P* |
| Non-SIL | 39 | 3 | 1 |  |  |
| LSIL | 84 | 1 | 0.15 | 0.002-2.028 | 0.070 |
| HSIL | 14 | 5 | 4.64 | 0.76-32.89 | 0.039 |
| CC | 8 | 15 | **24.37** | 4.93-151.12 | 0.0000 |
|  |  |  |  |  |  |
| Non-SIL | 39 | 3 | 1 |  |  |
| HSIL and CC | 22 | 20 | **11.82** | 2.92-66.96 | 0.0000 |
|  |  |  |  |  |  |
| Non-SIL and LSIL | 123 | 3 | 1 |  |  |
| HSIL and CC | 22 | 20 | **37.27** | 9.57-205.21 | 0.0000 |
|  | Methylated +  Unmethylated  n | Hypermethylated  n |  |  |  |
| Non-SIL | 56 | 3 | 1 |  |  |
| CC | 36 | 15 | **7.77** | 1.97-44.04 | 0.0006 |
| OR odd ratio, CI confidence interval, CC cervical cancer, HSIL high-grade squamous intraepithelial lesion, LSIL low-grade squamous intraepithelial lesion, Non-SIL negative for squamous intraepithelial lesion; Significant values for associations are indicated in bold. | | | | | |

| Table S5. Methylation status of MYOD1 and risk of cervical lesion | | | | | |
| --- | --- | --- | --- | --- | --- |
|  | Unmethylated  n | Hypermethylated  n | OR | CI | *P* |
| Non-SIL | 55 | 1 | 1 |  |  |
| LSIL | 102 | 1 | 0.54 | 0.006-43.08 | 0.659 |
| HSIL | 20 | 4 | 11 | 0.98-551.15 | 0.011 |
| CC | 27 | 9 | **18.33** | 2.27-817.58 | 0.0005 |
|  |  |  |  |  |  |
| Non-SIL | 55 | 1 | 1 |  |  |
| HSIL and CC | 47 | 13 | **15.21** | 2.09-657.87 | 0.0010 |
|  |  |  |  |  |  |
| Non-SIL and LSIL | 157 | 2 | 1 |  |  |
| HSIL and CC | 47 | 13 | **21.71** | 4.59-201.57 | 0.0000 |
|  | Methylated +  Unmethylated  n | Hypermethylated  n |  |  |  |
| Non-SIL | 59 | 1 | 1 |  |  |
| CC | 42 | 9 | **12.64** | 1.61-563.20 | 0.0034 |
| OR odd ratio, CI confidence interval, CC cervical cancer, HSIL high-grade squamous intraepithelial lesion, LSIL low-grade squamous intraepithelial lesion, Non-SIL negative for squamous intraepithelial lesion; Significant values for associations are indicated in bold. | | | | | |

| Table S6. Methylation status of SOX17 and risk of cervical lesion | | | | | |
| --- | --- | --- | --- | --- | --- |
|  | Unmethylated  n | Hypermethylated  n | OR | CI | *P* |
| Non-SIL | 40 | 2 | 1 |  |  |
| LSIL | 104 | 1 | 0.19 | 0.003-3.84 | 0.140 |
| HSIL | 21 | 1 | 0.95 | 0.015-19.31 | 0.969 |
| CC | 19 | 10 | **10.52** | 1.90-104.39 | 0.0010 |
|  |  |  |  |  |  |
| Non-SIL | 40 | 2 | 1 |  |  |
| HSIL and CC | 40 | 10 | 5 | 0.96-49.02 | 0.0306 |
|  |  |  |  |  |  |
| Non-SIL and LSIL | 144 | 3 | 1 |  |  |
| HSIL and CC | 40 | 10 | **18.94** | 3.56-172.14 | 0.0000 |
|  | Methylated +  Unmethylated  n | Hypermethylated  n |  |  |  |
| Non-SIL | 57 | 2 | 1 |  |  |
| CC | 41 | 10 | **6.95** | 1.35-67.47 | 0.0065 |
| OR odd ratio, CI confidence interval, CC cervical cancer, HSIL high-grade squamous intraepithelial lesion, LSIL low-grade squamous intraepithelial lesion, Non-SIL negative for squamous intraepithelial lesion; Significant values for associations are indicated in bold. | | | | | |

| Table S7. Methylation status of MGMT and risk of cervical lesion | | | | | |
| --- | --- | --- | --- | --- | --- |
|  | Unmethylated  n | Hypermethylated  n | OR | CI | *P* |
| Non-SIL | 55 | 1 | 1 |  |  |
| LSIL | 105 | 1 | 0.52 | 0.006-41.86 | 0.6443 |
| HSIL | 16 | 9 | **30.94** | 3.63-1379.3 | 0.0000 |
| CC | 36 | 1 | 1.52 | 0.02-122.06 | 0.765 |
|  |  |  |  |  |  |
| Non-SIL | 55 | 1 | 1 |  |  |
| HSIL and CC | 52 | 10 | **10.58** | 1.39-467.09 | 0.0074 |
|  |  |  |  |  |  |
| Non-SIL and LSIL | 160 | 2 | 1 |  |  |
| HSIL and CC | 52 | 10 | **15.38** | 3.09-146.74 | 0.0000 |
|  | Methylated +  Unmethylated  n | Hypermethylated  n |  |  |  |
| Non-SIL | 59 | 1 | 1 |  |  |
| CC | 52 | 1 | 1.13 | 0.014-90.55 | 0.9294 |
| OR odd ratio, CI confidence interval, CC cervical cancer, HSIL high-grade squamous intraepithelial lesion, LSIL low-grade squamous intraepithelial lesion, Non-SIL negative for squamous intraepithelial lesion; Significant values for associations are indicated in bold. | | | | | |

| Table S8. Methylation status of RASSF1A and risk of cervical lesion | | | | | |
| --- | --- | --- | --- | --- | --- |
|  | Unmethylated  n | Hypermethylated  n | OR | CI | *P* |
| Non-SIL | 59 | 1 | 1 |  |  |
| LSIL | 102 | 1 | 0.58 | 0.007-46.16 | 0.6971 |
| HSIL | 21 | 1 | 2.8 | 0.03-224.18 | 0.4540 |
| CC | 47 | 2 | 2.5 | 0.12-150.57 | 0.4433 |
|  |  |  |  |  |  |
| Non-SIL | 59 | 1 | 1 |  |  |
| HSIL and CC | 68 | 3 | 2.6 | 0.201-138.9 | 0.3964 |
|  |  |  |  |  |  |
| Non-SIL and LSIL | 161 | 2 | 1 |  |  |
| HSIL and CC | 68 | 3 | 3.5 | 0.39-43.14 | 0.1448 |
|  | Methylated +  Unmethylated  n | Hypermethylated  n |  |  |  |
| Non-SIL | 59 | 1 | 1 |  |  |
| CC | 49 | 1 | 1.2 | 0.015-96.1 | 0.8963 |
| OR odd ratio, CI confidence interval, CC cervical cancer, HSIL high-grade squamous intraepithelial lesion, LSIL low-grade squamous intraepithelial lesion, Non-SIL negative for squamous intraepithelial lesion. | | | | | |

| Table S9. Methylation status of CDH13 and risk of cervical lesion | | | | | |
| --- | --- | --- | --- | --- | --- |
|  | Unmethylated  n | Hypermethylated  n | OR | CI | *P* |
| Non-SIL | 58 | 1 | 1 |  |  |
| LSIL | 101 | 1 | 0.57 | 0.007-45.84 | 0.693 |
| HSIL | 25 | 1 | 2.32 | 0.028-185.18 | 0.546 |
| CC | 43 | 1 | 1.348 | 0.016-107.67 | 0.833 |
|  |  |  |  |  |  |
| Non-SIL | 58 | 1 | 1 |  |  |
| HSIL and CC | 68 | 2 | 1.705 | 0.086-102.34 | 0.662 |
|  |  |  |  |  |  |
| Non-SIL and LSIL | 159 | 2 | 1 |  |  |
| HSIL and CC | 68 | 2 | 2.338 | 0.165-32.733 | 0.387 |
|  | Methylated +  Unmethylated  n | Hypermethylated  n |  |  |  |
| Non-SIL | 59 | 1 | 1 |  |  |
| CC | 50 | 1 | 1.18 | 0.014-94.171 | 0.907 |
| OR odd ratio, CI confidence interval, CC cervical cancer, HSIL high-grade squamous intraepithelial lesion, LSIL low-grade squamous intraepithelial lesion, Non-SIL negative for squamous intraepithelial lesion. | | | | | |
