# Supplementary figures and images for: The CpG island methylator phenotype increases the risk of high-grade squamous intraepithelial lesions and cervical cancer
Source: Clin Epigenetics. 2022 Jan 6;14:4. doi: 10.1186/s13148-021-01224-0 (PMC8740093; doi:10.1186/s13148-021-01224-0)

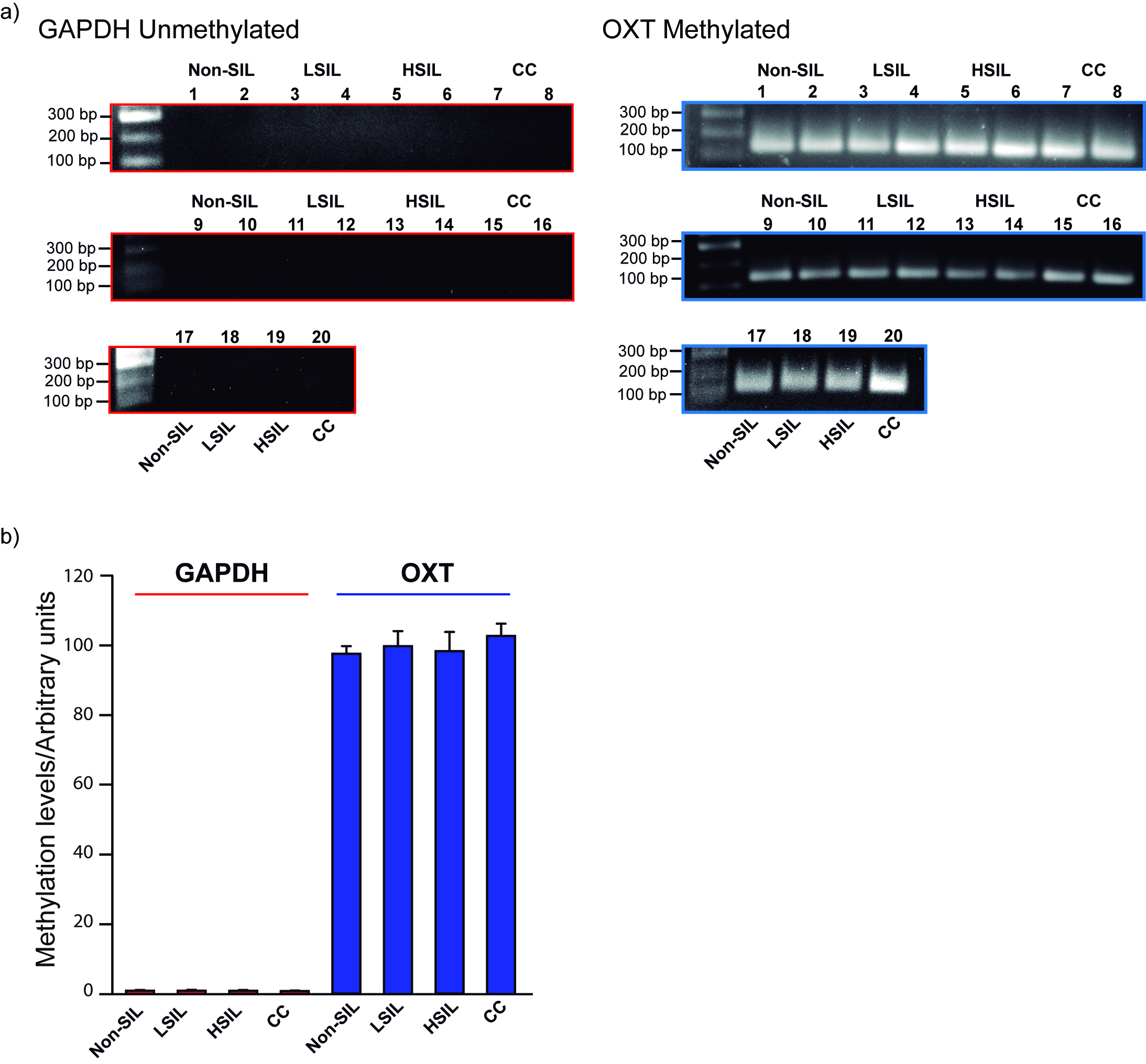

Supplement: Supplementary file 2 — Additional file 2: Fig. S1. Analysis of the methylation levels of control genes in cervical tissue. Methylation was analyzed in 5 samples negative for squamous intraepithelial lesions (non-SILs), 5 low-grade squamous intraepithelial lesions (LSILs), 5 high-grade squamous intraepithelial lesions (HSILs) and 5 cervical cancers (CCs). a) MSP products in agarose gels for methylated GAPDH (168 bp) and methylated OXT (131 bp). b) Densitometry analysis from GAPDH and OXT methylation. The data are presented as mean ± standard deviation at 5 samples in each group. [file 13148_2021_1224_MOESM2_ESM.tif]

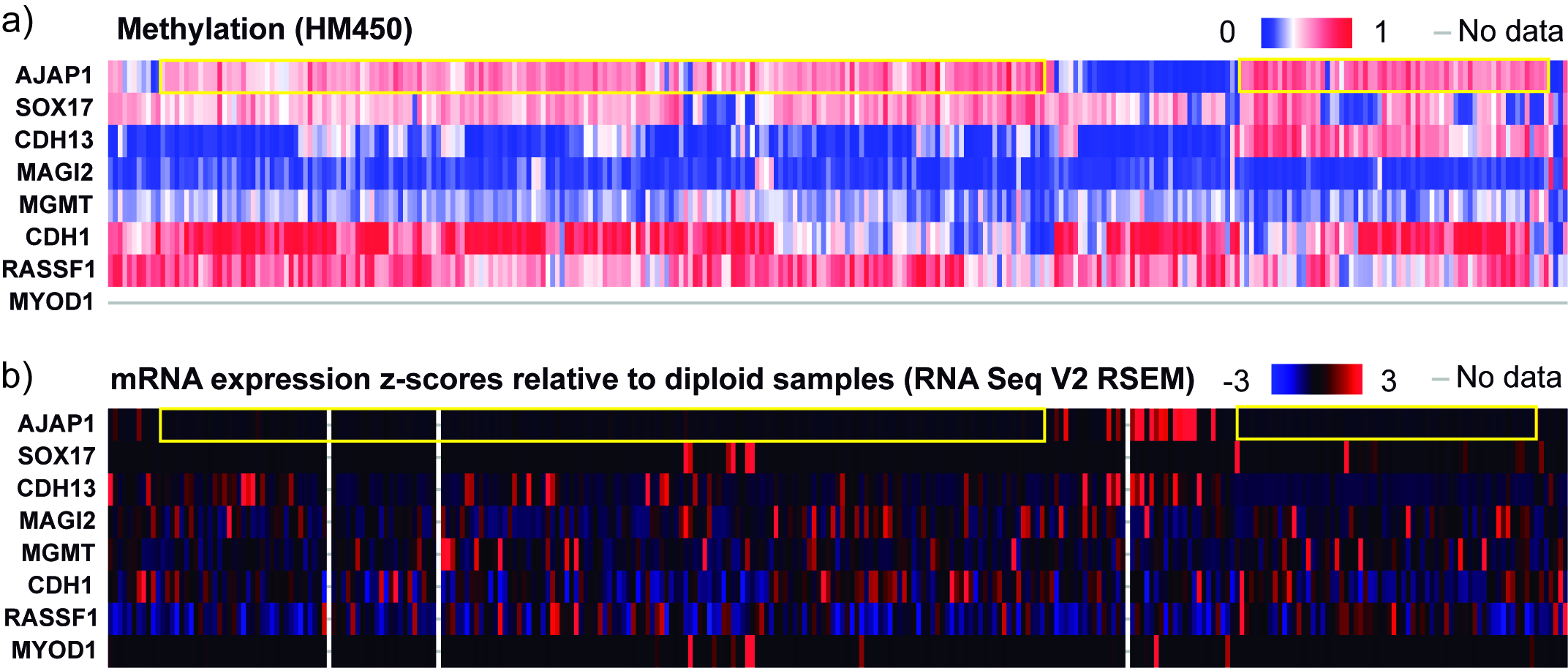

Supplement: Supplementary file 3 — Additional file 3: Fig. S2. Methylation and expression heatmaps of AJAP1, SOX17, CDH13, MAGI2, MGMT, CDH1, RASSF1, and MYOD1 genes in public database (Cervical Squamous Cell Carcinoma and Endocervical Adenocarcinoma, TCGA, Firehose Legacy). a) Methylation data of 309 patients. b) Expression level of analyzed genes. The yellow box shows a representative patient’s group for AJAP1 gene with high methylation levels and low expression levels. [file 13148_2021_1224_MOESM3_ESM.tif]

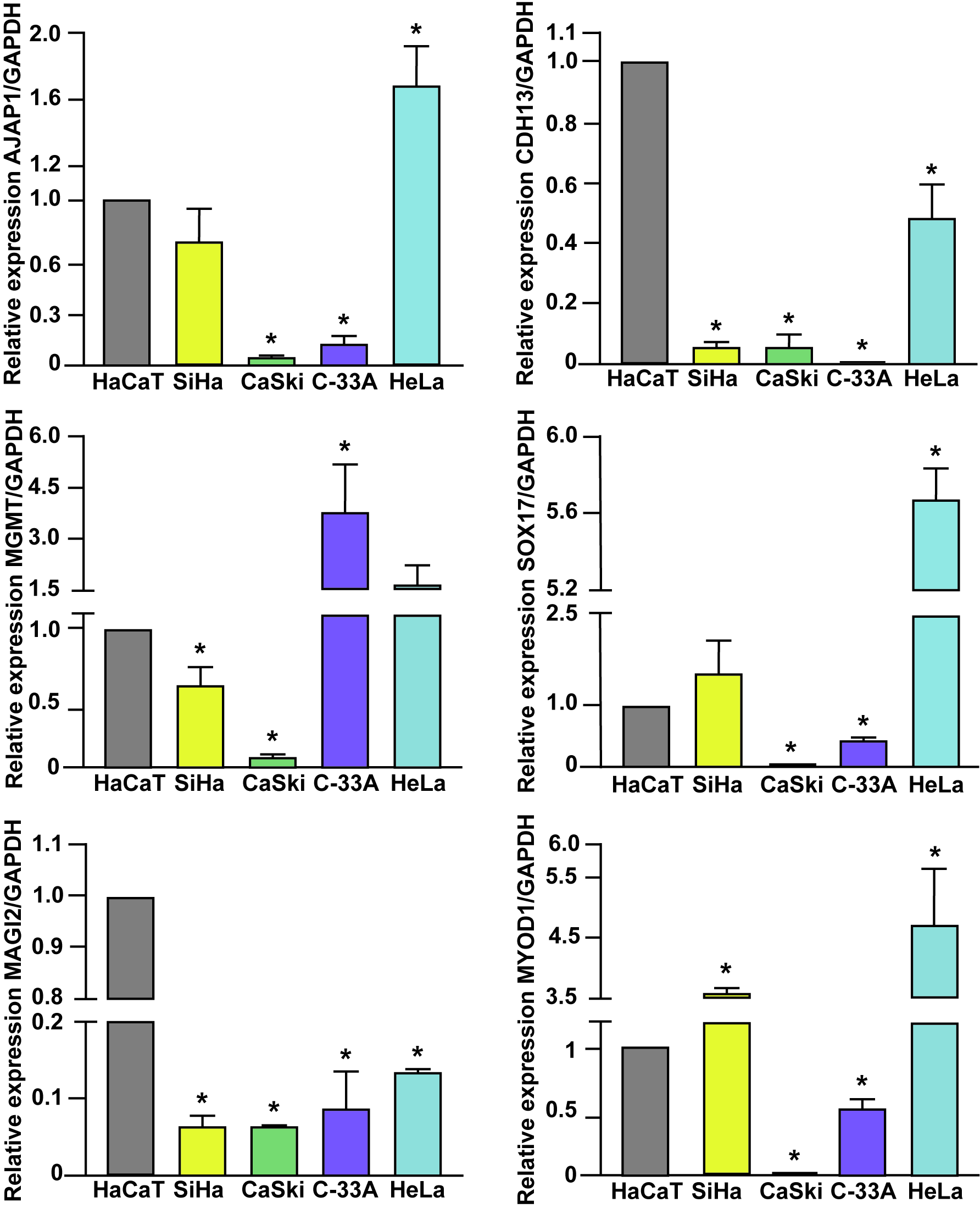

Supplement: Supplementary file 4 — Additional file 4: Fig. S3. Analysis of the expression levels of the AJAP1, CDH13, MAGI2, SOX17, MGMT, and MYOD1 genes in cervical cancer cell lines. The dates are presented as the fold change in cancer cell line relative to HaCaT cell line. *p<0.05 [file 13148_2021_1224_MOESM4_ESM.tif]

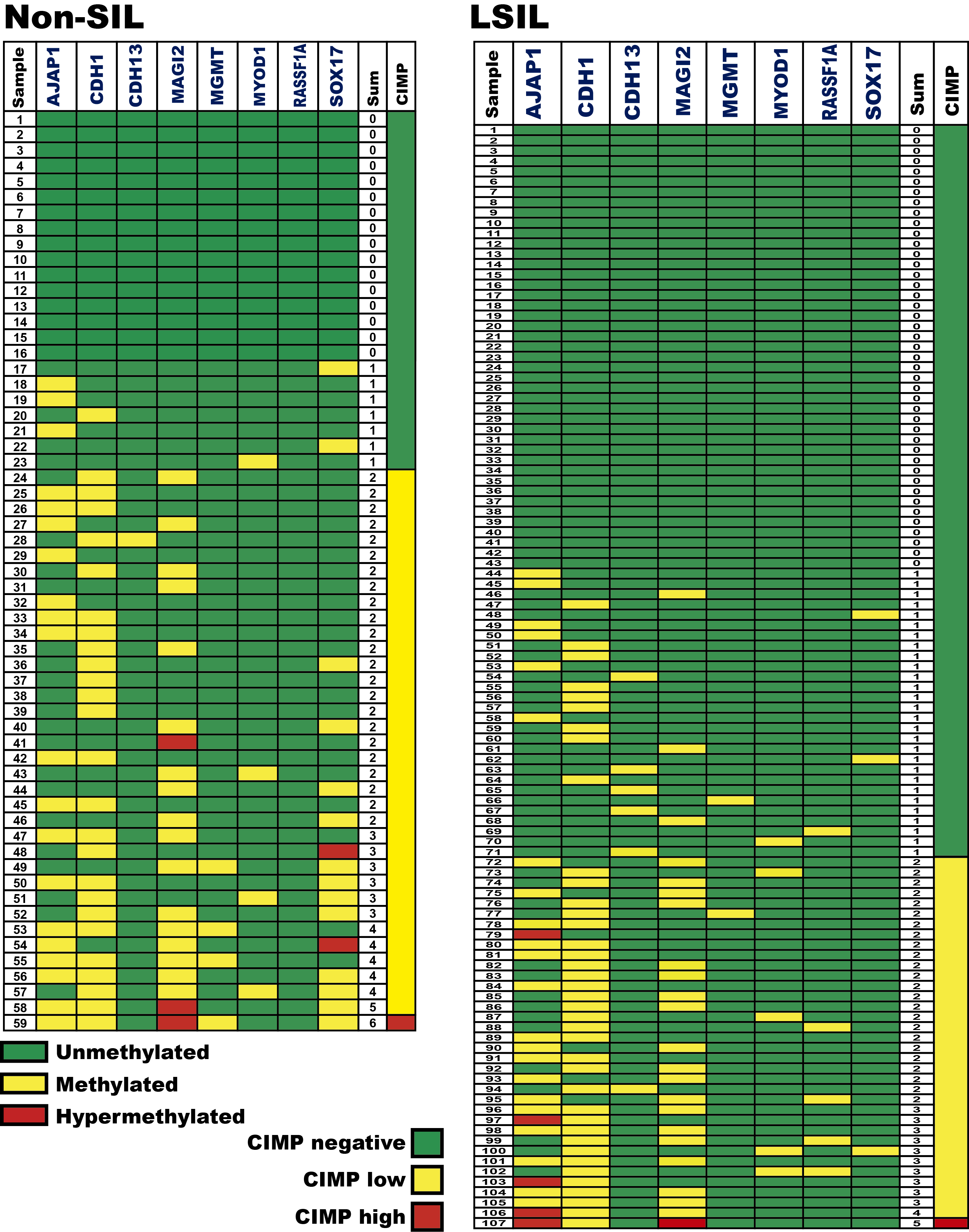

Supplement: Supplementary file 5 — Additional file 5: Fig. S4. Analysis of the methylation levels of the AJAP1, CDH1, CDH13, MAGI2, MGMT, MYOD1, RASSF1A and SOX17 genes in each of the non-SIL and LSIL samples. The sum of arbitrary values of methylation and CIMP status is shown. [file 13148_2021_1224_MOESM5_ESM.tif]

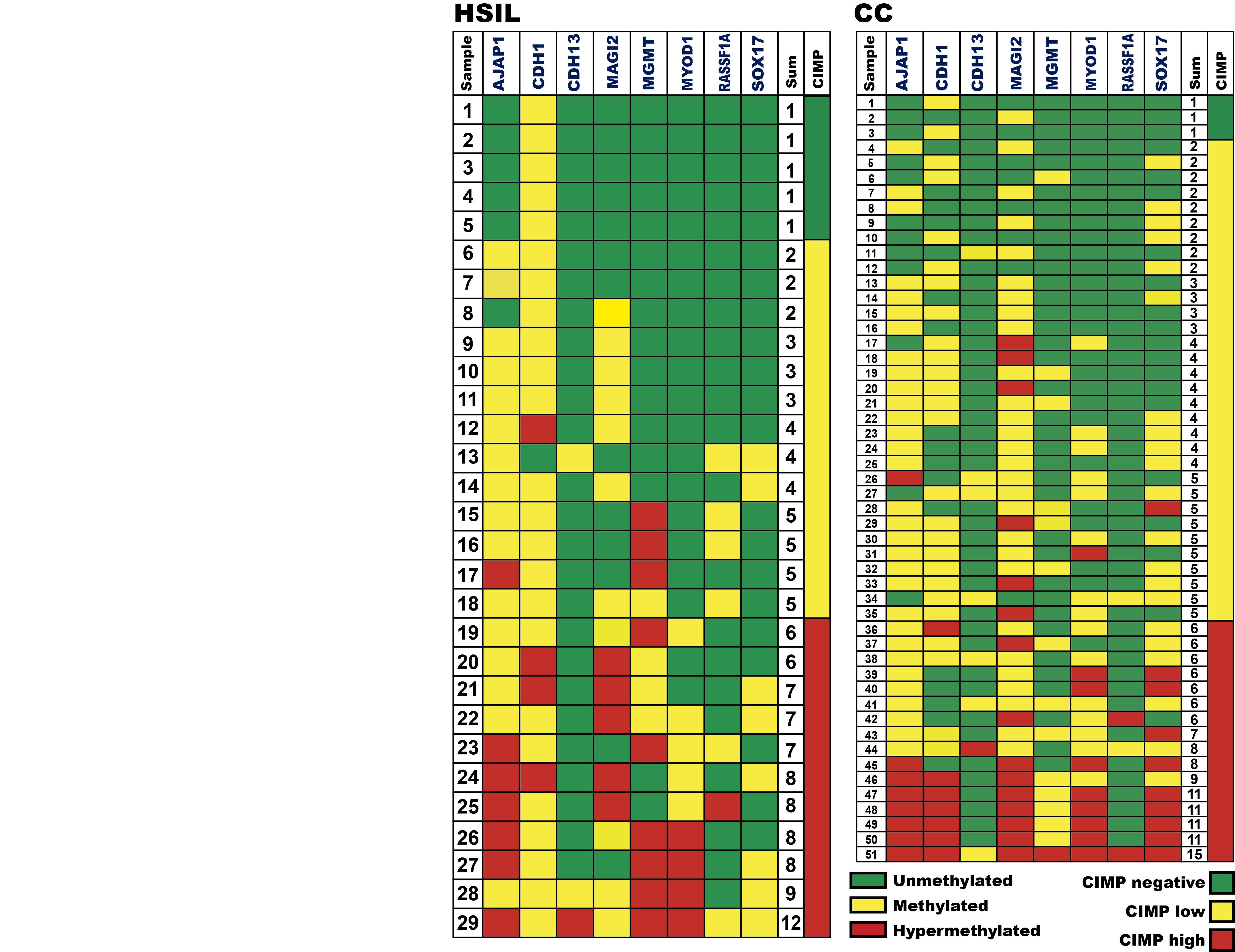

Supplement: Supplementary file 6 — Additional file 6: Fig. S5. Analysis of the methylation levels of the AJAP1, CDH1, CDH13, MAGI2, MGMT, MYOD1, RASSF1A and SOX17 genes in each of the HSIL and CC samples. The sum of arbitrary values of methylation and CIMP status is shown. [file 13148_2021_1224_MOESM6_ESM.tif]

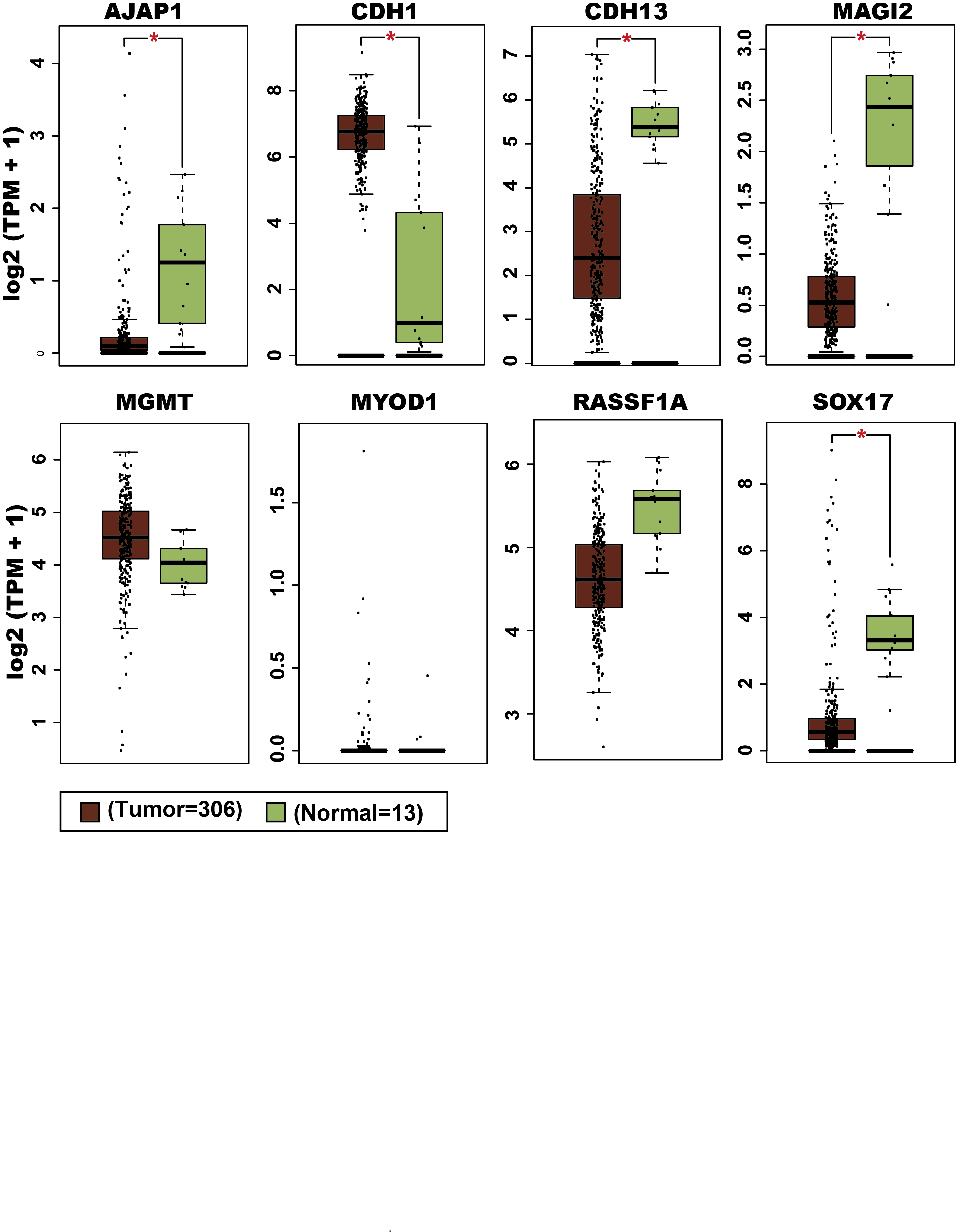

Supplement: Supplementary file 7 — Additional file 7: Fig. S6. Expression of analyzed genes in TCGA dataset. The mRNA levels of analyzed genes were investigated in normal and cancer samples. Cancer included cervical squamous cell carcinoma and endocervical adenocarcinoma tumor samples. *p<0.05 [file 13148_2021_1224_MOESM7_ESM.tif]
